# Effects of mentoring on self-reflection and competence in Final year medical students’ internal medicine rotation

**DOI:** 10.1371/journal.pone.0331057

**Published:** 2025-09-02

**Authors:** Markus B. Heckmann, Christine Mages, Daniel Finke, Christoph Reich, Ailís Ceara Haney, Caroline Riedel, Till Johannes Bugaj, Norbert Frey, Ann-Kathrin Rahm

**Affiliations:** 1 Department for Cardiology, Angiology, and Pneumology, Heidelberg University Hospital, Heidelberg, Germany; 2 German Centre for Cardiovascular Research: DZHK, Partner site Heidelberg, Mannheim, Germany; 3 Center for cardiac, vascular, and preventive medicine, ATOS Klinik, Heidelberg, Germany; 4 Department of General Internal Medicine and Psychosomatics, Heidelberg University Hospital, Heidelberg, Germany; King Abdulaziz University Faculty of Medicine, SAUDI ARABIA

## Abstract

**Background:**

Final year medical students (FYMS) in Germany transfer theoretical knowledge to clinical practice. However, there is a deficiency in structured guidance and feedback and self-reflective practices are often neglected. This study aims to investigate the impact of three mentoring strategies on cultivating self-reflection and introspection in FYMS and to assess competence during routine activities.

**Methods:**

Final year medical students (*n* = 46) completing a mandatory internal medicine rotation at Heidelberg University Hospital were randomized to either passive mentoring or active mentoring (split into structured and unstructured sessions). Across an 8-week rotation, we assessed the influence of mentoring styles on self-reflection and introspection (SRIS) at the beginning and at the end of the rotation. Changes in SRIS were compared by Mann Whitney U tests. Translated, adapted and newly generated questionnaires were analysed calculating cronbach’s alpha and optimized using an item-drop analysis.

**Results:**

Key findings include: a general reluctance to initiate mentor-mentee relationships (1 out of 15 in the passive group sought meetings, *p* < 0.0001), a propensity for FYMS to undervalue their competence compared to external evaluations (3.6 vs. 4.5 **p* *< 0.0001), a tendency for female FYMS to have higher perceived competence (PCS) than males, with means of 3.8 vs. 3.3 (*p* = 0.0021), and a measurable improvement in introspection (SRIS-IN) across all participants by the program’s end, regardless of the mentoring style received (median change on scale: 0.25, *p* = 0.0016). However, the mentoring intervention itself revealed no significant impact on self-reflection.

**Conclusions:**

Active mentoring had little to no effect on self-reflection and insight of final year medical students in Germany in the setting of an 8-week rotation. Interventional learning studies were well accepted, and participation rate was high. However, form completion rates were low. Future studies should focus on increasing form completion rates through additional incentives.

## Introduction

In various countries and health systems, the final year (FY) of medical education is a distinctive and crucial phase, where students undergo practical training in hospitals or medical institutions. In Germany, medical students in their FY (“Praktisches Jahr”, PJ) are required to complete clinical rotations in three core areas: Internal medicine, Surgery, and an elective field of their choice. This phase is characterized by hands-on experience, allowing students to apply their mainly theoretical knowledge in real-world clinical settings under the supervision of experienced physicians. The FY serves as a bridge between academic study and professional practice, providing students with the opportunity to develop their clinical skills, engage in patient care, and gain a deeper understanding of the medical profession before obtaining their full medical license. Medical students frequently feel there is a lack of guidance, supervision and feedback [1–4].

**Fig 1:**
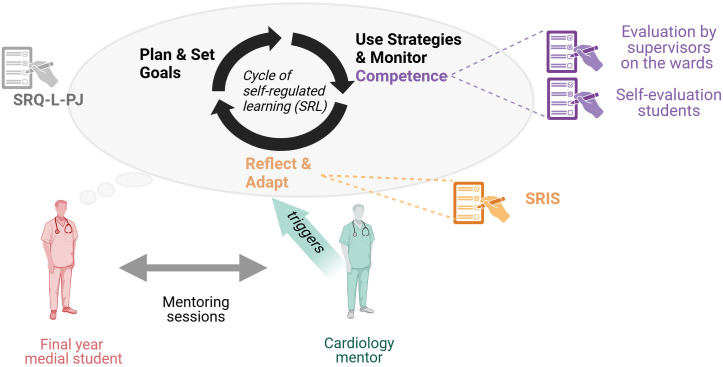
Conceptual framework for mentoring, self-reflection, self-regulated learning (SRL) and competence in our study concept. Initially, students completed the Learning Self-Regulation Questionnaire (SRQ-L-PJ) adapted for final-year students. The Self-Reflection and Insight Scale (SRIS) was administered at both the start and conclusion of the rotation. The timeline of the study can be found in Fig 2. Figure designed with BioRender.

The FY represents the crucial step to develop self-reflection and methods for life-long learning before starting a “life-long” career as a physician [3,5–7]. Life-long learning is a fundamental goal stated in Germany´s NKLM2.0 (the German National Competence-based Learning Objectives Catalogue for Undergraduate Medical Education) and has already been incorporated within the CanMEDS Roles [8]. Mentoring and support provision during this critical period is an under-researched area and in a German survey 42% of final year medical students (FYMS) reported not having a mentor or teaching coordinator to report to in case of problems [4,7]. Mentoring in the context of this study is a developmental partnership between an experienced physician and a less experienced medical student. The mentor provides guidance, support, and knowledge to the mentee, fostering professional growth, skill acquisition, and personal development (see Fig 1) [9].

Mentoring during FY has been shown to allow for individually tailored learning of clinical practical skills and to promote learning success mainly through feedback and individual learning support and a sense of belonging [2,9,10]. Students who received mentoring or feedback were indeed able to better improve in some competencies than students without mentoring and feedback [3], where competencies are defined in our study as an integrated set of knowledge, skills, and attitudes that enables a FY medical student to perform tasks and responsibilities in accordance with the expected standards.

Through guidance and feedback, mentors help mentees set learning objectives, reflect on their progress, and develop strategies to achieve their goals and seeking feedback for self-improvement [11]. Therefore, mentorship not only imparts technical skills but also fosters reflective thinking [9], which is crucial for lifelong learning [12] including the development of self-reflection and self-regulated learning (SRL) (see Fig 1).

Self-reflection, defined as an intentional process by which medical students and professionals engage in a critical examination of their own thoughts, actions, and experiences, represents the basis to identify personal knowledge and performance gaps and to set learning goals. Self-reflection has been associated with an increase in self-esteem and performance benefits [13,14].

Self-regulated learning (SRL) is defined as an approach whereby medical students assume responsibility for managing their own educational trajectory. This entails establishing personal learning objectives, implementing strategies to attain them, monitoring progress, and modifying techniques as necessary [15]. SRL is essential to professional learning and practice across disciplines. However, the literature provides limited insights into how medical educators could support trainees’ strategic decisions for learning goals [16]. In our view, this happens when medical educators become active mentors.

The aim of this study was to evaluate the effect of one-to-one mentoring on final-year medical students’ self-reflection using the Self-Reflection and Insight Scale (SRIS) [17], and both perceived (PCS) and externally assessed clinical competence scales (CS). Outcomes were assessed using validated instruments (SRIS, SRQ-L-PJ, PCS, CS) administered at baseline, midterm, and the end of the 8-week rotation. Secondary analyses explored potential associations with demographic variables, such as gender and age, to ensure a thorough understanding of potential confounders The Self-Regulated Learning Questionnaire (SRLQ) was adapted specifically for FYMS [17] and assessed at the beginning of the study to determine the baseline status in SRL. Our primary hypothesis was that active mentoring increases self-reflection and introspection and impacts self-experienced and also perceived competence [18].

## Methods

### Study design

This study was designed as an exploratory, randomized, controlled educational intervention using a mixed-methods framework, situated within the conceptual domain of mentoring and self-regulated learning in health professions education. Following recommendations by Ringsted et al. for educational research, we began by framing our intervention within a conceptual and theoretical framework informed by current literature on mentoring, self-reflection, and adult learning theories [18]. FYMS undergoing a mandatory internal medicine rotation at the Department of Cardiology and Heidelberg University Hospital were offered to enroll in a study investigating the effect of a mentoring program on self-reflection. FYMS were recruited in an introductory lecture for FYMS before their internal medicine rotation. In this lecture we routinely highlighted the need for life-long learning including a famous quotation from William Osler on pride and flagged this trait as another potential risk amongst doctors.

“In these days of aggressive self-assertion, when the stress of competition is so keen and the desire to make the most of oneself so universal, it may seem a little old-fashioned to preach the necessity of humility, but I insist …” [19]

**Fig 2:**
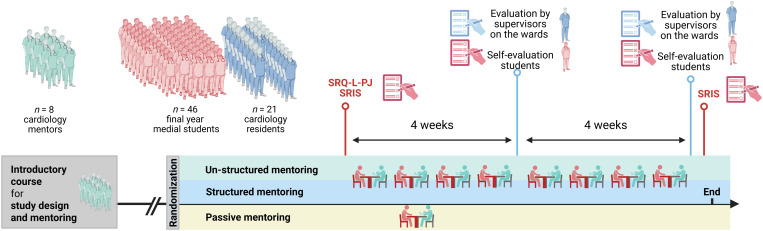
Study design. Final-year medical students were randomly assigned to active or passive mentoring during a mandatory internal medicine rotation. Active mentoring was categorized into structured and unstructured interviews. Initially, students completed the Learning Self-Regulation Questionnaire (SRQ-L-PJ) adapted for final-year students. The Self-Reflection and Insight Scale (SRIS) was administered at both the start and conclusion of the rotation. Midterm and post-rotation evaluations of perceived and actual competence in fulfilling standard medical duties on the ward were conducted by both students and their supervising residents. Figure designed with BioRender.

All FYMS who volunteered were randomly assigned to a mentor and a resident. Mentors were either cardiology fellows or trained cardiologists while residents oversaw a ward under the supervision of a cardiology attending. Upon written informed consent, Student-mentor partnerships were randomized into three intervention groups: (1) passive mentoring, (2) active mentoring with structured conversations, and (3) active mentoring with unstructured conversations (see Fig 2).

FYMS, mentors and residents were included in the study after written informed consent from 1^st^ May 2021 till 22^nd^ March 2022.

### Mentor preparation and guidelines

All cardiology mentors received a 30-minute orientation session. This session outlined the study purpose, mentoring expectations, and use of group-specific mentoring guides based on best practices derived from AMEE Guide No. 167: “Mentorship in Health Professions Education” [20]. The training emphasized psychological safety, fostering reflective learning, and balancing support with appropriate challenge.

Mentors in the **active mentoring – unstructured group** were tasked with initiating weekly contact with their mentees. They were encouraged to engage in informal dialogue focused on helping the student reflect on and derive personal meaning from their clinical experiences, without a predefined script or structure. The goal was to allow space for naturally evolving, mentee-led discussions, recognizing the value of emergent, situated learning described in communities of practice.

Mentors in the **active mentoring – structured group** followed a detailed mentoring guideline (see Supplemental Material S1 Text), structured around weekly meetings. Each session included: (1) reflection on daily routines and learning experiences; (2) structured self-reflection including SWOT analysis; and (3) goal setting and planning for the coming week. This design was grounded in AMEE recommendations that advocate clear developmental goals and intentional scaffolding of mentee learning. Mentors in this arm acted as developmental facilitators, focusing on enhancing self-reflection, insight, and competence through a structured dialogic process.

**Passive mentoring** was defined as a minimal-intervention condition. Mentors in this group were instructed to send a brief introductory email to their assigned mentee at the start of the rotation, informing them that they were available if needed. No further contact was initiated by the mentor unless the student reached out. This approach was chosen to simulate a common clinical environment where mentorship is available but not actively structured

### Randomization

Block randomization was implemented using in-house scripting in R to allocate students evenly across the three groups.

### Questionnaires

All instruments and their psychometric properties are provided in the Supplementary Materials. The conceptual basis for assessing self-regulated learning was derived from self-determination theory, while self-reflection was conceptualized as a critical metacognitive process for developing clinical competence. In this study, student participants were required to complete three questionnaires at specific intervals: the initial questionnaire was distributed within the first three days of the 8-week rotation, followed by a second questionnaire after four weeks, and a final one at the eighth-week mark, marking the conclusion of the study. The initial questionnaire gathered data on participants’ demographics and included two key components: The Self-Reflection and Introspection Survey (SRIS), which was translated into German, is a 5 point Likert scale type questionnaire asking subjects the extent to which they agree or disagree with 20 statements [21,22]. The responses to each question are scored on a scale of one to five with one equating to “strongly disagree” and five to “strongly agree”. The statements relate to three domains of insight: recognition of the need for reflection, the process of engaging in reflection and the presence of insight. The second component was a translated and adapted Self-Regulated Learning Questionnaire (SRQ-L-PJ) [17,23,24]. All questionnaires were translated into German.

The second questionnaire included participant characteristics and introduced an evaluation of their perceived clinical competence (PCS). The final questionnaire after 8 weeks repeated the participant characteristics, reassessed perceived clinical competence (PCS), and included the SRIS once more.

Additionally, resident physicians were tasked with evaluating the students’ clinical competencies (CS) at the four and eight-week intervals, mirroring the content of the students’ questionnaire. The evaluation was not based on video recordings but derived from 2–4 weeks of experience of the supervising physician working with the student. Typical CS like writing patient notes, admitting patients to the ward (physical examination etc.), presenting cases to colleagues, planning therapy and checking-up on the progress of therapies. Perceived medical skills assessment items were derived from the entrustable professional activities (EPAs) outlined in the graduate profile of the NKLM 2.0, the German National Competence-based Learning Objectives Catalogue for Undergraduate Medical Education [25].

### Exploratory factor analysis and content validity

The questionnaire development was based on structured framework proposed by Artino et al., which guided the overall development process [26]. Content validity was established prior to data collection. Two experienced medical educators independently reviewed all translated and adapted questionnaire items (SRIS, SRQL-PJ, PCS, CS) for linguistic clarity, cultural appropriateness, and alignment with the intended constructs. Their feedback was used to refine item wording, resolve translation ambiguities, and ensure conceptual equivalence to the original instruments. Consensus was reached on all included items.

To assess the construct validity of the translated and adapted questionnaires used in this study, we conducted a series of exploratory factor analyses (EFA) using principal axis factoring with oblimin rotation, allowing for correlated latent constructs [S1–S4 Figs]. All analyses were performed in R (version 4.3.0) using the *psych* (version 2.5.6) package. Prior to EFA, data suitability was verified using Bartlett’s test of sphericity and the Kaiser-Meyer-Olkin (KMO) measure of sampling adequacy. Parallel analysis and scree plot inspection guided the determination of the number of factors to extract. Factor loadings ≥ 0.40 were interpreted as meaningful, loadings ≥ 0.30 were included in the EFA graphs.

### Data management

To ensure timely responses, reminders were sent every other day. For the administration of these questionnaires and data collection, a Lime Survey server was utilized. Data management was facilitated through custom scripting in R (version 4.3.0), which included the generation of tokens, server access via the ‘limer’ package, randomization of participants, and automation of email reminders.

Mentors had to fill out online reports on the duration (in minutes) and location of mentoring sessions every week and were reminded by E-Mail alerts to hold the sessions. Detailed content of the mentoring sessions was not assessed.

All questionnaires are available in the supplementary material.

### Ethics statement

This study adhered to the ethical guidelines outlined in the Declaration of Helsinki and received approval from the Heidelberg University ethics committee (Reference: S-671/2020). All participants gave written informed consent for participation in this study.

### Statistical analysis

Statistical analyses were performed using R (version 4.3.0).

The primary hypothesis, whether active mentoring increases self-reflection and introspection, was tested comparing the SRIS-IN and SRIS-SR values of the two groups (active and passive mentoring groups) using a Wilcoxon-Mann-Whitney test. A Spearman method was used to correlate values derived from a Likert scale. Uni- and multivariate regression models were created using a negative binominal regression analysis with the help of the MASS package (version 7.3–60.0.1). A Chi-squared test was used to compare count data and contingency tables, when normal distribution could be assumed a student’s T-test was performed for multiple comparison and analysis of variance was performed (ANOVA). Non-parametric data – such as data derived from Likert scales – were compared using a Wilcoxon-Mann-Whitney test for two groups comparison or using a Kruskal-Wallis test for multiple groups. A *P* value below 0.05 was considered significant. Participants tables were created using the tableone package (version 0.13.2). *P*-values reported in the tables are not intended for hypothesis testing, but they were merely calculated and reported in accordance with current academic practices and should be used in an explorative nature.

Univariate and multivariate models were created using the MASS package (version 7.3–60.0.1). A negative binominal regression model was chosen to create the generalized models. Theta was determined using score and information iterations until both converged.

## Results

### Trial participants and characteristics

Demographic data from students and mentors were examined to account for potential co-variation in univariate and multivariate regression models. A total of 46 students, 21 residents, and 8 mentors participated in this trial. Only about a third of students (17/56; 37%) and residents (7/21; 33%) were female, while half of the mentors were female (4/8; 50%). There were no participants who attributed a non-binary sex or gender to themselves. The students’ mean age was 28 ± 4 years (mean ± standard deviation), the residents’ mean age 31 ± 3 years, and the mentors’ mean age 32 ± 3 years. 15 Students were enrolled in the passive mentoring arm and 31 students were enrolled in the active mentoring arm, of which 16 were assigned to the structured and 15 students to the unstructured interview protocol.

### Study completion

4 students (9%) did not fill out any questionnaire, 7 students (15%) only filled out the baseline questionnaire, 10 students filled out baseline and the first midterm evaluation (22%), and 25 students completed the whole study (54%) despite several timely e-mail alerts. The numbers did not vary significantly between the passive and active mentoring group (53% and 55% respectively, *p* = 0.9235). In a univariate negative binominal regression model female sex (OR 1.24 [0.83;1.84, 95% CI], *p* = 0.2775) was positively – but not significantly – associated with higher study completion levels, while age, controlled regulation (in which behavior is driven by external pressures, rewards, punishments, or obligations rather than by internal desires or values), autonomous regulation (where actions are driven by personal values or intrinsic interest), and the mentoring group (passive versus active) did not show a relevant association (OR 1.01 [0.96;1.05] *p* = 0.8273, OR 1.03 [0.76;1.41] *p* = 0.8303, OR 1.05 [0.75, 1.47], *p* = 0.7899, and OR 1.01 [0.67;1.55] **p* *= 0.9561, respectively). All students’ characteristics are summarized in Table 1.

**Table 1:** Student participants’ characteristics.

	Passive Mentoring	Active Mentoring	*p*-Value
Total N	15	31	
Female sex (%)	5 (33.3)	12 (38.7)	0.977
Age (mean (SD))	27.79 (2.79)	28.54 (4.91)	0.585
Stage of training (%)			0.592
1. tertial	9 (64.3)	15 (53.6)	
2. tertial	2 (14.3)	8 (28.6)	
3. tertial	3 (21.4)	5 (17.9)	
Interview time (mean (SD))	8.60 (13.77)	39.77 (45.09)	0.012
Same sex mentoring	8 (53.3)	16 (51.6)	1.000
Completion stage (%)			0.924
no questions answered	1 (6.7)	3 (9.7)	
only baseline	3 (20.0)	4 (12.9)	
baseline and midterm	3 (20.0)	7 (22.6)	
baseline midterm and final	8 (53.3)	17 (54.8)	
External review (%)			0.491
no external assessment	8 (53.3)	11 (35.5)	
midterm	4 (26.7)	10 (32.3)	
midterm and final	3 (20.0)	10 (32.3)	
Desired internal medicine residency	6 (42.9)	14 (50.0)	0.913
SRLQ scales			
autonomous (median [IQR])	4.40 [4.20, 4.75]	4.20 [3.60, 4.60]	0.197
controlled (median [IQR])	3.33 [3.21, 3.79]	3.33 [3.00, 3.67]	0.840
SRIS-Scales			
Introspection: SRIS-IN			
baseline (median [IQR])	3.94 [3.47, 4.34]	3.62 [3.38, 4.16]	0.512
final (median [IQR])	4.19 [3.94, 4.59]	3.94 [3.38, 4.38]	0.171
difference (median [IQR])	0.31 [0.03, 0.69]	0.19 [−0.12, 0.56]	0.510
Self-Reflection: SRIS-SR			
baseline (median [IQR])	3.25 [2.94, 4.23]	3.75 [3.46, 4.00]	0.316
final (median [IQR])	4.00 [3.23, 4.15]	3.79 [3.60, 4.15]	0.719
difference (median [IQR])	−0.17 [−0.25, 0.06]	0.00 [−0.23, 0.40]	0.630
Perceived competence scale			
midterm (median [IQR])	3.46 [2.81, 3.81]	3.42 [2.48, 3.77]	0.762
final (median [IQR])	3.71 [3.35, 3.96]	3.67 [3.15, 4.29]	0.648
Competence scale			
midterm (median [IQR])	4.35 [3.92, 4.92]	4.60 [4.34, 5.00]	0.597
final (median [IQR])	4.40 [4.17, 4.51]	4.43 [4.22, 5.00]	0.531
Preparedness for duty			
perceived			
midterm (median [IQR])	4.00 [3.25, 5.00]	4.00 [3.00, 4.00]	0.109
final (median [IQR])	4.00 [3.25, 4.75]	4.00 [4.00, 4.75]	0.817
externally assessed			
midterm (median [IQR])	5.00 [4.25, 5.00]	5.00 [5.00, 5.00]	0.107
final (median [IQR])	5.00 [5.00, 5.00]	5.00 [5.00, 5.00]	0.418

### Weekly mentoring

Weekly mentoring sessions were generally only conducted when initiated by the mentor. Only one out of 15 students who were subjected to passive mentoring asked for a meeting once, whereas all students in the active mentoring group had conversations with a mentor (*p* < 0.0001). The median time per mentoring was 20 [12–30 IQR] minutes and mentoring were most frequently conducted in-person in the cafeteria (40%) or the doctor’s room on the ward (26%), followed by a conversation on the phone (15%).

### Factor structure and validity evidence

The self-reflection and insight scale (SRIS) demonstrated a two-factor structure consistent with the underlying dimensions of self-reflection and insight (see S1 Fig). Bartlett’s test indicated adequate factorability (χ²(190) = 736.47, p < 0.001), and the overall KMO was 0.79. Parallel analysis supported a two-factor solution, which explained 46.4% of the variance (PA1: 25.9%, PA2: 20.5%). Items aligned closely with their theoretical dimensions; reflective items (e.g., Items 7, 10, 12, 15, 16, 18, 19) loaded on PA1, while insight-related items (e.g., Items 4, 9, 11, 14, 17, 20) loaded on PA2.

SRQL-PJ scale suggested a two-factor solution corresponding to the theoretical dimensions of autonomous regulation and controlled regulation. The KMO was 0.68 and Bartlett’s test confirmed factorability (χ²(66) = 214.54, p < 0.001). Together, the two factors explained 45.1% of the variance (PA1: 31.8%, PA2: 13.3%). Items corresponding to autonomous motivation (e.g., Items 1, 4, 8, 9, 11) loaded on the first factor, while controlled items (e.g., Items 5, 6, 10) loaded on the second. However, the distinction between the two factors emerged only marginally above the threshold in the parallel analysis, indicating limited empirical discriminability between the constructs (see S2 Fig). This limitation was reflected in the cross-loading of individual items, such as item 3, which conceptually represents controlled regulation but loaded partially on the autonomous factor. These findings suggest that although the theoretical structure is preserved, the empirical distinction between autonomous and controlled regulation was not fully supported in this sample of German final-year medical students. This may be due to conceptual overlap, cultural nuances in translation, or the specific educational context. Therefore, the results should be interpreted cautiously when assigning items strictly to distinct subscales.

EFA of the perceived competence scale (PCS) revealed a unidimensional structure, with all items loading on a single factor (see S3 Fig). The KMO was excellent at 0.88, and Bartlett’s test was significant (χ²(66) = 448.65, p < 0.001). This factor explained 50.6% of total variance. Negatively worded items loaded inversely (e.g., Items 3, 4, 9), suggesting consistent construct representation across item directions.

The competence scale (CS) also exhibited a single-factor structure (see S4 Fig), accounting for 56.8% of the variance. KMO was 0.74, and Bartlett’s test was significant (χ²(55) = 293.07, p < 0.001). Items demonstrated strong factor loadings ranging from 0.52 to 0.89, supporting the scale’s use as a cohesive assessment of clinical competence by supervising residents. EFA of the

### Analysis of questionnaires and internal reliability

With a Cronbach’s alpha of 0.87 (0.81;0.93 95% CI, *n* = 42) and 0.86 (0.80;0.92 95% CI, **n* *= 42) for SRIS-IN and SRIS-SR at baseline, respectively, the SRIS questionnaire showed a good internal reliability (see S1 Table). Similar values were calculated for the final questionnaire (SRIS-IN: 0.87 (0.81;0.93), SRIS-SR: 0.89(0.84; 0.95)). These values compared well to the values published (0.87 and 0.91, respectively) [23].

The translated and adapted SRQ-L-PJ questionnaire performed comparably weak, especially the controlled regulation scale 0.62 (0.44;0.79, 95% CI, *n* = 42). An item-drop analysis revealed that the question “I will follow the instructions and suggestions of my resident doctor because I want to avoid conflicts with him/her” reduced Cronbach’s alpha from 0.67 to 0.62. After dropping this item, an item-drop analysis revealed that dropping the question “I will actively participate in patient care because I would be proud of myself if I successfully contribute” further increased Cronbach’s alpha to 0.73 (0.60;0.86) (see S2 Table). The SRQ-L-PJ autonomous regulation scale showed comparable values to previously published data (0.71 [0.57;0.85]).

On a 5-item Lickert scale—results of questionnaire by group autonomous regulation (4.40 [4.20, 4.75]) and (4.20 [3.60, 4.60]) active mentoring group. The difference was not statistically significant (p = 0.1970) controlled (median [IQR]) 3.33 [3.21, 3.79] and 3.33 [3.00, 3.67] active mentoring group 0.840. This might reflect the weak construct separation mentioned above.

Our developed 12-item scale for perceived competence (PCS, S3 Table) showed a good internal reliability at midterm (Cronbach’s alpha: 0.92 [0.88; 0.96, 95% CI n = 34) and at the end of rotation (0.91 [0.86; 0.96, n = 28]). Similarly, the 10-item competence scale (CS, S4 Table) used by the residents showed good internal reliability at midterm (0.89 [0.82; 0.95], n = 14) and at the end of rotation (0.95 (0.91; 0.99), n = 11). As mentioned in the methods section, the questions were derived from the entrustable professional activities (EPAs) outlined in the German graduate profile of the NKLM 2.0 [25].

### Active mentoring and self-reflection

**Fig 3:**
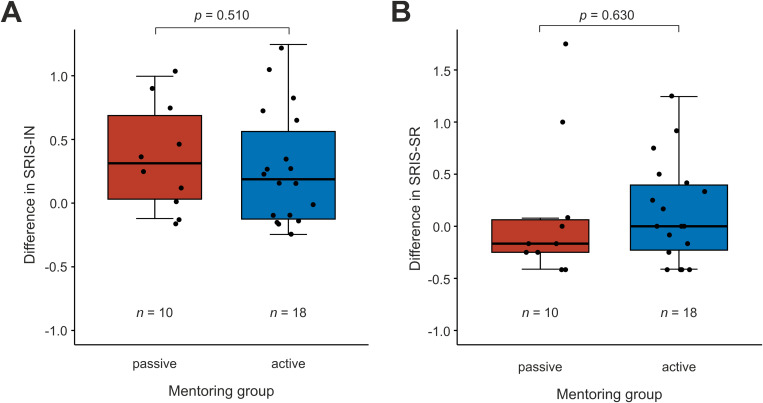
Self-Reflection and Introspection Scale (SRIS). **A** Differences SRIS Introspection (SRIS-IN) between baseline and end of rotation by group. **B** Differences SRIS self-reflection (SRIS-SR) between baseline and end of rotation by group. A Mann-Whitney-U test was used to test for statistical significance. Only students who finished the study were included in this analysis.

Overall self-reflection measured by SRIS-SR did not change over the course of the 8-week rotation 0 (−0.25–0.35, IQR) *p* = 0.5963 while insight, measured by SRIS-IN, increased in all groups SIRS-IN 0.25 (−0.13–0.66, IQR) *p* = 0.0016. Active mentoring did not lead to a significant increase in self-reflection or introspection. The baseline SRIS-IN and SRIS-SR values were 3.94 [3.47–4.34 IQR] and 3.25 [2.94–4.23] in the passive group as well as 3.62 [3.38–4.16] (*p* = 0.5120) and 3.75 [3.46–4.00] (*p* = 0.3160) in the active group. The change in SRIS-IN and SRIS-SR was 0.31 [0.03–0.69] and −0.17 [−0.25–0.06], respectively, in the passive group as well as 0.19 [−0.12, 0.56] and 0.00 [−0.23, 0.40], respectively, in the active group. The change was not significantly different between the groups (*p* = 0.510 and *p* = 0.630, respectively, see Fig 3).

### Competence and self-reflection

**Fig 4:**
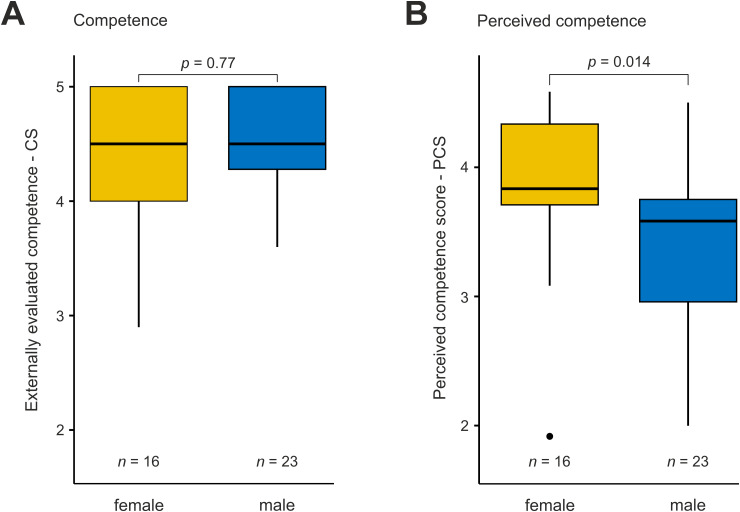
Competence and perceived competence by sex. In our study population competence scores as rated by the supervising physician were the same amongst female and male students **(A)**, while perceived competence was lower in males than in females **(B)**.

**Fig 5:**
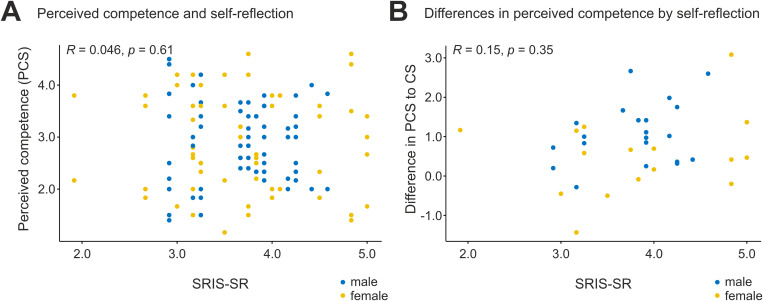
Competence and Perceived Competence. **A** Perceived Competence Scores (PCS) are plotted against Self-Reflection scores (SRIS-SR). Higher PCS values denote greater self-assessed competence, while higher SRIS-SR values suggest more extensive self-reflection. **B** The difference between externally rated Competence (CS) and Perceived Competence (PCS) is graphed against SRIS-SR scores. Positive CS-PCS values indicate instances where external assessments of competence are higher than self-perceived competence. With increasing self-reflection, PCS may significantly undershoot CS, resulting in a triangular distribution pattern.

We also evaluated the student’s competence in everyday clinical work as self-reflection influences learning and, thus, eventually competence. This was done by self-evaluation (perceived competence) and evaluations by supervising residents (competence). Perceived competence was lower (PCS 3.6 [2.9–4.0], IQR]) as the evaluated competence measured by the competence scale (CS 4.5 [4.0–5.0], *p* < 0.0001). Female students rated their competence (PCS) better than male students (3.8 [3.5–4.3] vs. 3.3 [2.5–3.6], *p* = 0.0141) while external competence ratings did not differ (4.5 [4.0–5.0], 4.5 [4.3–5.0], *p* = 0.7719, see Fig 4). There was a tendency towards underestimating one’s competence with increased SIRS-SR scores, see Fig 5. This becomes evident when the PCS is subtracted from the externally evaluated CS and plotted to SRIS-SR (see Fig 5).

### Regression models for the endpoints form completion and competence

In the univariate and multivariate analysis of controlled regulation, autonomous regulation, female sex, active mentoring and age did not show any statistical association with form completion (see S5 Table).

In the univariate analysis for competence, controlled regulation showed a trend toward significance with an OR of 1.16, while autonomous regulation also indicated a similar trend with an OR of 1.22 (see S5 Table). However, female sex, active mentoring and age were not significantly associated with competence. In the multivariate analysis, controlled regulation, autonomous regulation, female sex and active mentoring also remained non-significant.

## Discussion

Self-reflected, life-long learning is a central tenet of the German National Competence-based Learning Objectives Catalogue for Undergraduate Medical Education (NKLM 2.0), which emphasizes that graduates should continuously evaluate their professional performance and identify their individual learning needs (“The graduate reviews their professional knowledge and actions and continuously identifies their own learning needs in the sense of a lifelong learning process.”). While reflective learning has gained attention in recent years within medical education, the empirical evidence connecting reflective capacity to improved clinical outcomes remains limited [25]. Nonetheless, several studies have demonstrated that reflection supports deeper learning, enhances self-awareness, and facilitates meaningful professional development among medical trainees [20,26,27]. Reflection enables learners to bridge experience with theory, develop professional identity, and critically engage with clinical complexities [18,20,28]. However, students typically do not naturally adopt reflective learning habits and thus require guidance from teachers in this regard [29,30]

### Does self-reflection and insight can be increased by active mentoring?

In this study, we examined whether mentoring could serve as a viable intervention to support self-reflection and introspection in final-year medical students (FYMS). While we observed a significant improvement in students’ introspection (SRIS-IN) scores over the course of their 8-week internal medicine rotation, our results suggest that this change occurred irrespective of whether students received active or passive mentoring. In other words, the mentoring style—structured, unstructured, or passive—did not significantly influence the trajectory of self-reflection or insight.

Several factors may explain these findings. First, the short duration of the mentoring intervention may have limited its potential impact. Mentoring, particularly when aimed at fostering reflective habits, is a developmental process that typically unfolds over time. Our 8-week intervention may not have provided sufficient continuity or depth to yield measurable gains beyond those resulting from the clinical experience itself. Second, FYMS were embedded within teams led by residents who likely provided informal teaching and role modeling—introducing uncontrolled but potentially beneficial mentoring experiences across all groups. Third, although mentors were trained and given guidelines aligned with best practices (e.g., AMEE Guide No. 167), the intensity and quality of implementation may have varied across individuals.

Interestingly, even minimal exposure to structured clinical environments appeared to promote introspective growth. This suggests that while formal mentoring structures may be helpful, they are not the sole determinants of reflective development. However, our findings also caution against over-relying on passive mentoring: in our study, very few students in the passive arm actively sought mentorship, despite reporting a desire for such support. Only 7% of students with an assigned passive mentor used this resource. This dissonance highlights a possible mismatch between perceived need and help-seeking behavior, and reinforces the importance of proactive mentoring strategies in future educational designs.

### Associations between learning regulation, mentoring, and outcomes

Our analysis found no significant associations between controlled regulation, autonomous regulation, sex, age, or active mentoring with form completion or competence. Although univariate analyses showed trends toward significance for controlled regulation (OR = 1.16, p = 0.0699) and autonomous regulation (OR = 1.22, p = 0.0580) with competence, these did not reach statistical significance and were not sustained in multivariate models. These results suggest that, within our study’s scope, neither the type of regulation nor mentoring style significantly impacted students’ engagement with the study (form completion, only 54% of study participants completed all questionnaires) or their competence. The absence of significant findings might be attributed to the limited sample size or the short duration of the mentoring intervention.

### Recommendations for future projects

To build on our findings, future mentoring interventions in medical education should consider several refinements:

1. **Extend Duration and Continuity**: Reflective habits require time to develop. Longitudinal mentoring relationships—ideally spanning multiple clinical rotations—may foster deeper engagement and transformation [18,20,27].
2. **Embed Mentoring in Structured Curricula**: Aligning mentoring with core curricular activities, such as portfolio reviews or regular reflective writing, may help integrate reflection into daily practice [18,20,28].
3. **Train Mentors More Intensively**: In line with AMEE guidance, mentor development programs should include instruction in coaching techniques, feedback delivery, and strategies to promote psychological safety [20,29,30].
4. **Encourage Mentee Activation**: Future interventions should include strategies to empower students to take a more active role in mentoring relationships, potentially supported by peer mentoring networks or speed mentoring models [31].
5. **Assess Mentoring Quality**: Beyond measuring presence or absence of mentoring, or time spent with mentoring sessions, qualitative assessments of the relationship quality, mentor behavior, and mentee engagement are essential to better understand the dynamics at play [32].
6. **Incorporate Reflective Assessment Tools**: Combining self-report questionnaires with richer data sources (e.g., structured learning diaries, mentor feedback, or narrative reflections) could yield more nuanced insights into reflective growth [33].

## Limitations

Despite its strengths as a randomized and conceptually grounded trial, this study has several limitations. Mentoring, particularly when aimed at fostering reflective habits, is a developmental process that typically unfolds over time. Our 8-week intervention may not have provided sufficient continuity or depth to yield measurable gains beyond those resulting from the clinical experience itself. Despite the short duration, significant improvement in introspection (SRIS-IN) was observed across all groups—suggesting the rotation itself may contribute to some reflective growth. Another major limitation is the low study completion rate of only 54%. Almost no student declined participation in this study and all were assigned a mentor. Interestingly, only half of the students filled out the short questionnaires. It seems that sending automated emails and reminders was not sufficient to help the participants contribute to research. Due to these limitations, a small effect of short-term mentoring on self-reflection and competence might be missed. Blinding was not feasible. Although students were not told their assigned group, the mentor’s activity gave it away, so students and mentors were not blinded to the intervention groups and many students noticed the difference between an active and passive mentoring style. Another limitation might be the voluntary nature of the study, as this entails at least some intrinsic or extrinsic motivation. In most medical education studies doing more teaching than less improves results and would not be specific to the teaching intervention. In the end (as expected) the group were the students needed to take the active part ended with no mentoring as a control group. Additionally comparison of multiple mentoring types was included into the study as we wanted to know which mentoring concept would be best added into clinical routine duties for the mentors.

While the two-factor structure of the SRQL-PJ aligns with the assumptions of self-determination theory, the factorial evidence indicates only partial separation of autonomous and controlled regulation. This may be due to contextual influences (e.g., the structure of clinical training during the final year), linguistic ambiguities in translation, or conceptual proximity between subtypes of motivation in this setting. Future validation studies should further investigate the item structure and consider cultural or educational adaptations to improve discriminant validity. Although Cronbach’s alpha was used to assess internal reliability, it was complemented by item-drop analysis and exploratory factor analysis to provide a more robust evaluation of the instrument’s psychometric properties, as detailed in the supplements

## Conclusion

Our study shows that active mentoring has little to no effect on self-reflection and insight of final year medical students in Germany in the setting of an 8-week rotation. Interventional learning studies were well accepted and participation rate was high, however, form completion rates were low. Future studies should focus on increasing form completion rates through additional incentives.

## Supporting information

S1 Table The SRIS. (DOCX)

S2 Table SRQ-L-PJ. (DOCX)

S3 Table Perceived competence scale (PCS). (DOCX)

S4 Table Competency Scale (CS). (DOCX)

S5 Table Regression models for the endpoints form completion and competence. (DOCX)

S1 Text Instructions to mentors. (DOCX)

S2 Text Supplementary Figure Legends. (DOCX)

S1 Fig EFA – SIRS. (TIF)

S2 Fig EFA – SRQL-PJ. (TIF)

S3 Fig EFA – PCS. (TIF)

S4 Fig EFA – CS. (TIF)

S1 File References. (DOCX)
