# Supplementary material for: Effects of mentoring on self-reflection and competence in Final year medical students’ internal medicine rotation
Source: PLoS One. 2025 Sep 2;20(9):e0331057. doi: 10.1371/journal.pone.0331057 (PMC12404468; doi:10.1371/journal.pone.0331057)
Supplement: S1 Table — (DOCX) [file pone.0331057.s001.docx]

**Supplementary Material**

**Questionnaires**

**STable 1.** The SRIS (Self-Reflection and Insight Scale) questionnaire assesses an individual's capacity for self-reflection and their level of insight.^1^ Questions marked with (R) are reverse-scored.

|  |  | **Scale** | **Start**  *n* = 42  Cronbach’s alpha | **Final**  *n* = 28  Cronbach’s alpha |
| --- | --- | --- | --- | --- |
|  | **Global** | SRIS-SR | 0.86 | 0.89 |
|  |  | SRIS-IN | 0.87 | 0.88 |
| **#** | **Questions: item-drop analysis** |  |  |  |
| 03 | I am usually aware of my thoughts. | SRIS-IN | 0.89 | 0.90 |
| 04 | I am often confused about the way that I really feel about things (R) | SRIS-IN | 0.85 | 0.86 |
| 06 | I usually have a very clear idea about why I have behaved in a certain way. | SRIS-IN | 0.87 | 0.88 |
| 09 | I’m often aware that I am having a feeling, but I often don’t quite know what it is (R). | SRIS-IN | 0.84 | 0.85 |
| 11 | My behavior often puzzles me (R). | SRIS-IN | 0.85 | 0.86 |
| 14 | Thinking about my thoughts makes me more confused (R). | SRIS-IN | 0.85 | 0.87 |
| 17 | Often I find it difficult to make sense of the way I feel about things (R). | SRIS-IN | 0.84 | 0.87 |
| 20 | I usually know why I feel the way I do | SRIS-IN | 0.85 | 0.86 |
| 01 | I don’t often think about my thoughts (R) | SRIS-SR | 0.86 | 0.91 |
| 02 | I am not really interested in analyzing my behavior (R) | SRIS-SR | 0.88 | 0.89 |
| 05 | It is important for me to evaluate the things that I do. | SRIS-SR | 0.87 | 0.89 |
| 07 | I am very interested in examining what I think about. | SRIS-SR | 0.84 | 0.87 |
| 08 | I rarely spend time in self-reflection (R). | SRIS-SR | 0.86 | 0.89 |
| 10 | I frequently examine my feelings. | SRIS-SR | 0.83 | 0.88 |
| 12 | It is important to me to try to understand what my feelings mean. | SRIS-SR | 0.84 | 0.88 |
| 13 | I don’t really think about why I behave in the way that I do (R). | SRIS-SR | 0.85 | 0.87 |
| 15 | I have a definite need to understand the way my mind works. | SRIS-SR | 0.83 | 0.89 |
| 16 | I frequently take time to reflect on my thoughts. | SRIS-SR | 0.85 | 0.88 |
| 18 | It is important to me to be able to understand how my thoughts arise. | SRIS-SR | 0.83 | 0.87 |
| 19 | I often think about the way I feel about things. | SRIS-SR | 0.84 | 0.88 |
