# Supplementary material for: Effects of mentoring on self-reflection and competence in Final year medical students’ internal medicine rotation
Source: PLoS One. 2025 Sep 2;20(9):e0331057. doi: 10.1371/journal.pone.0331057 (PMC12404468; doi:10.1371/journal.pone.0331057)
Supplement: S2 Table — (DOCX) [file pone.0331057.s002.docx]

**Supplementary Material**

**Questionnaires**

**STable 2. SRQ-L-PJ.** This questionnaire adapted from the SRQ-L^2^ questionnaire to the diverse motivations driving final-year medical students to participate in patient care during their Praktisches Jahr, ranging from personal and professional growth to external expectations and obligations. It takes into account the trade-off between intrinsic desires for improvement and skill acquisition, and the extrinsic pressures of departmental dynamics and mandatory program requirements.

|  |  | **Scale** | **Start**  *n* = 42  Cronbach’s alpha |
| --- | --- | --- | --- |
|  | **Global** | Auto | 0.71 |
|  |  | Contr | 0.73 |
| **#** | **Questions: item-drop analysis** |  |  |
| 01 | I will actively engage in patient care: Because caring for patients during the PJ (Praktisches Jahr) helps me become a better doctor. | Auto | 0.64 |
| 04 | I will actively engage in patient care: Because I believe it prepares me to manage a ward as a physician myself. | Auto | 0.62 |
| 11 | The reason why I engage more intensively in patient care is that it is challenging to solve clinical cases. | Auto | 0.65 |
| 12 | The reason why I engage more intensively in patient care is that I enjoy working with patients and want to improve in that area. | Auto | 0.66 |
| 08 | I will follow the instructions and suggestions of my ward doctor because he/she probably knows better how I can make the most of this time. | Auto | 0.74 |
| 03 | I will actively engage in patient care, as others might think poorly of me if I did not. | Contr | 0.56 |
| 09 | The reason why I engage more intensively in patient care: Because I finally want to show others what I can do medically. | Contr | 0.66 |
| 10 | The reason why I engage more intensively in patient care: Only because I have to. It's a MANDATORY rotation! | Contr | 0.67 |
| 06 | I will follow the instructions and suggestions of my resident because I want to leave a good impression in the department. | Contr | 0.78 |
| 07 | I will follow the instructions and suggestions of my resident because I can learn important skills much faster through him/her. | Contr | 0.62 |
|  |  |  |  |
|  | **items dropped** |  |  |
| 02 | I will actively engage in patient care, as I would be proud of myself if I contribute successfully. | Contr |  |
| 05 | I will follow the instructions and suggestions of my resident doctor because I want to avoid conflicts with the resident. | Contr |  |
