# Supplementary material for: Effects of mentoring on self-reflection and competence in Final year medical students’ internal medicine rotation
Source: PLoS One. 2025 Sep 2;20(9):e0331057. doi: 10.1371/journal.pone.0331057 (PMC12404468; doi:10.1371/journal.pone.0331057)
Supplement: S3 Table — (DOCX) [file pone.0331057.s003.docx]

**Supplementary Material**

**Questionnaires**

**STable 3. Perceived competence scale (PCS).** This questionnaire assesses the confidence of final-year medical students in their clinical abilities during the Praktisches Jahr. Questions marked with (R) are reverse-scored.

|  |  | **Midterm** | **Final** |
| --- | --- | --- | --- |
|  |  | *n* = 34 | *n* = 28 |
|  |  | Cronbach's alpha | Cronbach's alpha |
|  | **Global** | 0.92 | 0.92 |
| **#** | **Questions: item-drop analysis** |  |  |
| 01 | I feel confident that I could lead a ward as a resident. | 0.91 | 0.90 |
| 02 | I participate in the implementation of the therapeutic concept by regularly reviewing the patient list and considering what the patients still need. | 0.91 | 0.89 |
| 03 | With a good treatment plan, I could take care of every patient myself. (R) | 0.91 | 0.90 |
| 04 | I don't get very involved because it doesn't change anything anyway. (R) | 0.91 | 0.90 |
| 05 | I actively participate in the creation of therapy concepts. | 0.91 | 0.89 |
| 06 | I often think about the next steps for patients already at admission. | 0.91 | 0.90 |
| 07 | I often understand why patients receive a certain therapy. | 0.92 | 0.89 |
| 08 | I know why my patients are taking their medications. | 0.92 | 0.90 |
| 09 | I often don't understand the therapy concepts applied. (R) | 0.91 | 0.90 |
| 10 | I feel uncertain when admitting and presenting patients. | 0.91 | 0.90 |
| 11 | I understand the medical problem of the patients that I admit. | 0.92 | 0.92 |
| 12 | I can admit patients and communicate their cases in a structured manner. | 0.92 | 0.90 |
