# Supplementary material for: Effects of mentoring on self-reflection and competence in Final year medical students’ internal medicine rotation
Source: PLoS One. 2025 Sep 2;20(9):e0331057. doi: 10.1371/journal.pone.0331057 (PMC12404468; doi:10.1371/journal.pone.0331057)
Supplement: S4 Table — (DOCX) [file pone.0331057.s004.docx]

**Supplementary Material**

**Questionnaires**

**Supplementary Table 4: Competency Scale (CS).** This questionnaire filled out by residents assesses key skills of medical students, including medical report writing, patient care participation, case management reliability, therapeutic plan development and implementation, practical medical procedure proficiency, structured patient presentation, medical understanding, and overall dependability.

|  |  | **Final**  *n* = 14 | **Midterm**  *n* = 34 |
| --- | --- | --- | --- |
|  |  | Cronbach’s alpha | Cronbach’s alpha |
|  | **Global** | 0.95 | 0.89 |
|  | **Questions: Item-Drop-Analysis** |  |  |
| 01 | He/she can independently write a medical report. | 0.94 | 0.89 |
| 02 | He/she actively participates in patient care. | 0.94 | 0.86 |
| 03 | I can delegate cases to him/her and rely on him/her. | 0.94 | 0.89 |
| 04 | He/she can carry out meaningful therapeutic plans. | 0.94 | 0.85 |
| 05 | He/she can develop meaningful therapeutic plans. | 0.93 | 0.84 |
| 06 | He/she is proficient in practical medical activities (physical examination/ECG/blood draws/injections, etc.) | 0.94 | 0.90 |
| 07 | He/she can present patients in a structured manner during rounds. | 0.94 | 0.85 |
| 08 | He/she can admit patients and present them in a structured way. | 0.95 | NA |
| 09 | He/she understands the medical aspects of patient care. | 0.95 | 0.89 |
| 10 | I could rely on him/her | 0.94 | 0.86 |
|  |  |  |  |
