# Supplementary material for: Effects of mentoring on self-reflection and competence in Final year medical students’ internal medicine rotation
Source: PLoS One. 2025 Sep 2;20(9):e0331057. doi: 10.1371/journal.pone.0331057 (PMC12404468; doi:10.1371/journal.pone.0331057)
Supplement: S5 Table — (DOCX) [file pone.0331057.s005.docx]

**Supplementary Material**

**Questionnaires**

**STable 5: Regression models for the endpoints form completion and competence**

|  | **Univariate analysis** | ***p*** | **Multivariate analysis** | ***p*** |
| --- | --- | --- | --- | --- |
| **Form Completion** |  |  |  |  |
| Controlled regulation | 1.02 (0.76-1.38) | 0.9198 | 0.89 (0.59-1.35) | 0.5816 |
| Autonomous regulation | 1.15 (0.77-1.75) | 0.5019 | 1.23 (0.69-2.26) | 0.4888 |
| Female sex | 1.24 (0.83-1.84) | 0.2775 | 1.15 (0.75-1.77) | 0.5212 |
| Active mentoring | 1.01 (0.67- 1.55) | 0.9561 | 1.07 (0.70-1.67) | 0.7537 |
| Age | 1.01 (0.96-1.05) | 0.8273 | n.a. |  |
| **Competence** |  |  |  |  |
| Controlled regulation | 1.16 (0.99 -1.37) | 0.0699 | 1.07 (0.83-1.37) | 0.6192 |
| Autonomous regulation | 1.22 (0.99-1.51) | 0.0580 | 1.18 (0.86-1.65) | 0.3127 |
| Female sex | 1.08 (0.88-1.32) | 0.4419 | 0.99 (0.80-1.23) | 0.9338 |
| Active mentoring | 1.03 (0.83-1.29) | 0.764 | 0.99 (0.80-1.23) | 0.9338 |
| Age | 1.00 (0.97-1.02) | 0.7745 | n.a. |  |
