# Supplementary material for: Effects of mentoring on self-reflection and competence in Final year medical students’ internal medicine rotation
Source: PLoS One. 2025 Sep 2;20(9):e0331057. doi: 10.1371/journal.pone.0331057 (PMC12404468; doi:10.1371/journal.pone.0331057)
Supplement: S1 Text — (DOCX) [file pone.0331057.s006.docx]

**Supplementary Material**

# **SText1: Instructions to mentors**

**For mentors of mentees in group 1 (passive mentoring):**

Subject: New Mentee [FirstName LastName]

Hi [MentorFirstName],

You have a new mentee: [FirstName LastName] [StudentEmail]

Group: no conversation initiative

You only need to introduce yourself once and then wait for your mentee to approach you.

You can document your interviews at the following link: [Link]

Best regards, Your Seneca-Trial Team

**For mentors of mentees in group 2 (active structured mentoring):**

Subject: New Mentee [FirstName LastName]

Hi [MentorFirstName],

You have a new mentee: [FirstName LastName] [StudentEmail]

Group: structured conversations

In this group, you are asked to conduct structured conversations:

- 5 min. Daily routine on the ward (What are your tasks? How is your day structured?)

- 5 min. Self-reflection (How do you feel about your tasks? Is there a special learning opportunity given your current situation? What are your goals? What do you want to learn more about?)

- 10 min. Planning (How will you proceed to learn? What will you change or keep? How will you measure/control your learning success?)

You can document your interviews at the following link: [Link]

Best regards, Your Seneca-Trial Team

**For mentors of mentees in group 3 (active unstructured mentoring):**

Subject: New Mentee [FirstName LastName]

Hi [MentorFirstName],

You have a new mentee: [FirstName LastName] [StudentEmail]

Group: unstructured conversations

You are asked to contact your mentee once a week and talk to them in an informal and not goal-oriented manner.

You can document your interviews at the following link: [Link]

Best regards, Your Seneca-Trial Team

Note: `[FirstName LastName]`, `[MentorFirstName]`, `[StudentEmail]`, and `[Link]` are placeholders, which were filled with the actual names, emails, and links specific to each mentor-mentee pair.
