# Supplementary material for: Effects of mentoring on self-reflection and competence in Final year medical students’ internal medicine rotation
Source: PLoS One. 2025 Sep 2;20(9):e0331057. doi: 10.1371/journal.pone.0331057 (PMC12404468; doi:10.1371/journal.pone.0331057)
Supplement: S2 Text — (DOCX) [file pone.0331057.s007.docx]

**Supplementary Material**

**SText2: Supplementary Figure Legends**

**SFigure 1:** Exploratory factor analyses (EFA) for the translated self-reflection and insight scale (SRIS) questionnaire. **(A)** Parallel analysis scree plot showing eigenvalues from the actual data (blue triangles), simulated random data (dotted red line), and resampled data using bootstrapping (dashed red line). Two factors from the actual data clearly exceeded the simulated and resampled thresholds, supporting a two-factor solution. **(B)** Factor loading diagram of the final two-factor solution. Items SRIS Items 7, 10, 12, 15, 16, 18, and 19 loaded strongly on Factor 1 (PA1: Self-Reflection), while items 4, 9, 11, 14, 17, and 20 loaded on Factor 2 (PA2: Insight). Items with weaker (below 0.3) or cross-loadings were not connected. **Actual data**: eigenvalues calculated from the original correlation matrix. **Simulated dat**a: based on random data with no correlation (noise benchmark). **Resampled** data: bootstrap-derived eigenvalues preserving item structure.

**SFigure 2:** Exploratory factor analyses (EFA) for the translated Self-Regulated Learning Questionnaire adapted for final year medical students (FYMS) (SRQL-PJ) questionnaire. **(A)** Parallel analysis indicates a marginally acceptable two-factor structure. The second factor’s eigenvalue from actual data (blue triangle) slightly exceeds the resampled and simulated thresholds. (B) Items SRQL Items 1, 4, 7, 8, 9, 10, and 12 load on Factor 1 (PA1, interpreted as autonomous regulation), while items 2, 3, 5, and 6 load on Factor 2 (PA2, controlled regulation). SRQL Item 3 and 6 show cross-loadings. Loading for item 2 were below the 0.3 threshold. The factors reflect theoretical constructs, but the empirical separation is limited. **Actual data**: eigenvalues calculated from the original correlation matrix. **Simulated dat**a: based on random data with no correlation (noise benchmark). **Resampled** data: bootstrap-derived eigenvalues preserving item structure.

**SFigure 3:** Exploratory factor analyses (EFA) for the perceived clinical competence scale (PCS) adapted for final year medical students (FYMS). **(A)** Scree plot from parallel analysis indicates a clear unidimensional structure. Only the first actual eigenvalue (blue triangle) exceeds both resampled and simulated thresholds. **(B)** All PCS items load significantly on the single factor (PA1), including reversed-coded items (e.g., items 3, 4, and 7) with negative loadings. The PCS measures a coherent, single underlying construct of perceived clinical competence. **Actual data**: eigenvalues calculated from the original correlation matrix. **Simulated dat**a: based on random data with no correlation (noise benchmark). **Resampled** data: bootstrap-derived eigenvalues preserving item structure.

**SFigure 4:** Exploratory factor analyses (EFA) for the assessed clinical competence scale (CS) adapted for final year medical students (FYMS). **(A)** Parallel analysis strongly supports a one-factor solution, with only the first eigenvalue from actual data above the resampled/simulated lines.

**(B)** All 11 CS items load strongly on a single factor (PA1), indicating consistent assessment of externally observed clinical competence. The CS scale demonstrates excellent internal consistency and structural unidimensionality. **Actual data**: eigenvalues calculated from the original correlation matrix. **Simulated dat**a: based on random data with no correlation (noise benchmark). **Resampled** data: bootstrap-derived eigenvalues preserving item structure.
