# Supplementary figures and images for: Effects of mentoring on self-reflection and competence in Final year medical students’ internal medicine rotation
Source: PLoS One. 2025 Sep 2;20(9):e0331057. doi: 10.1371/journal.pone.0331057 (PMC12404468; doi:10.1371/journal.pone.0331057)

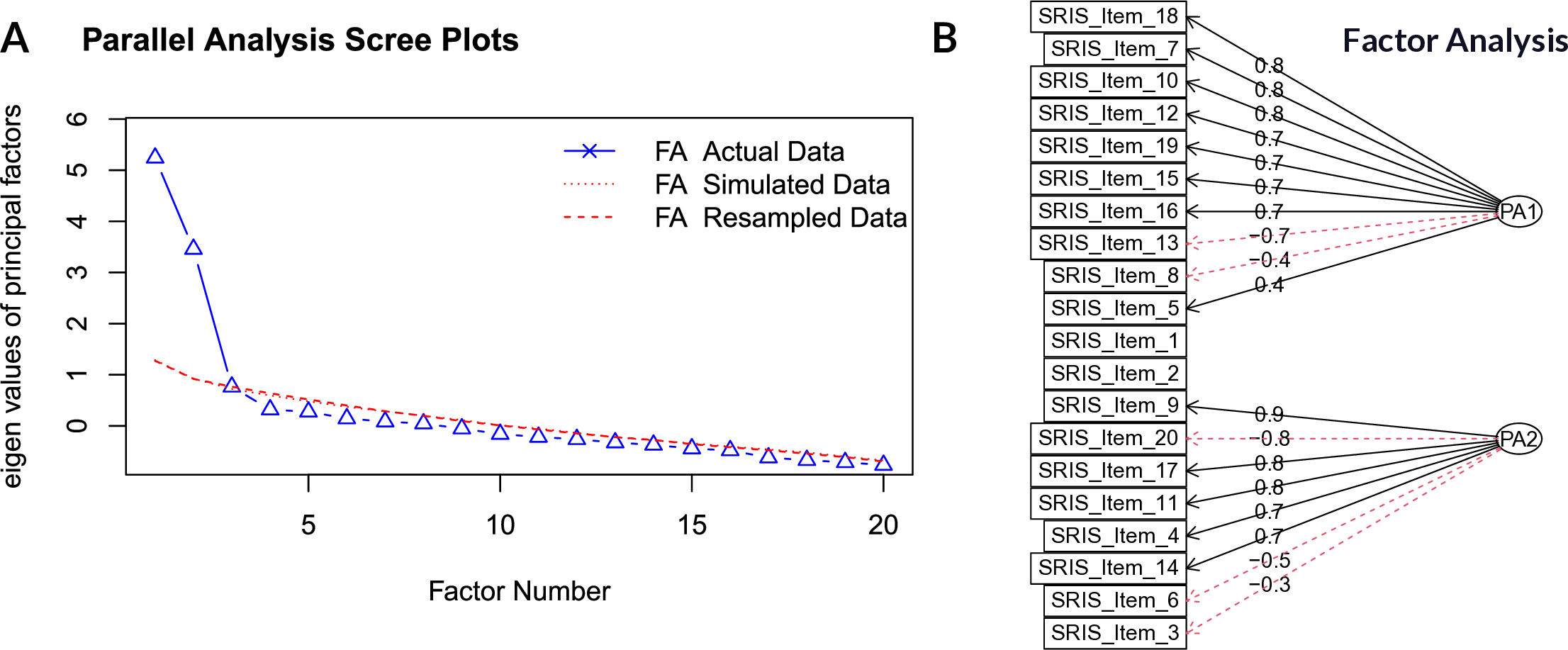

Supplement: S1 Fig — (TIF) [file pone.0331057.s008.tif]

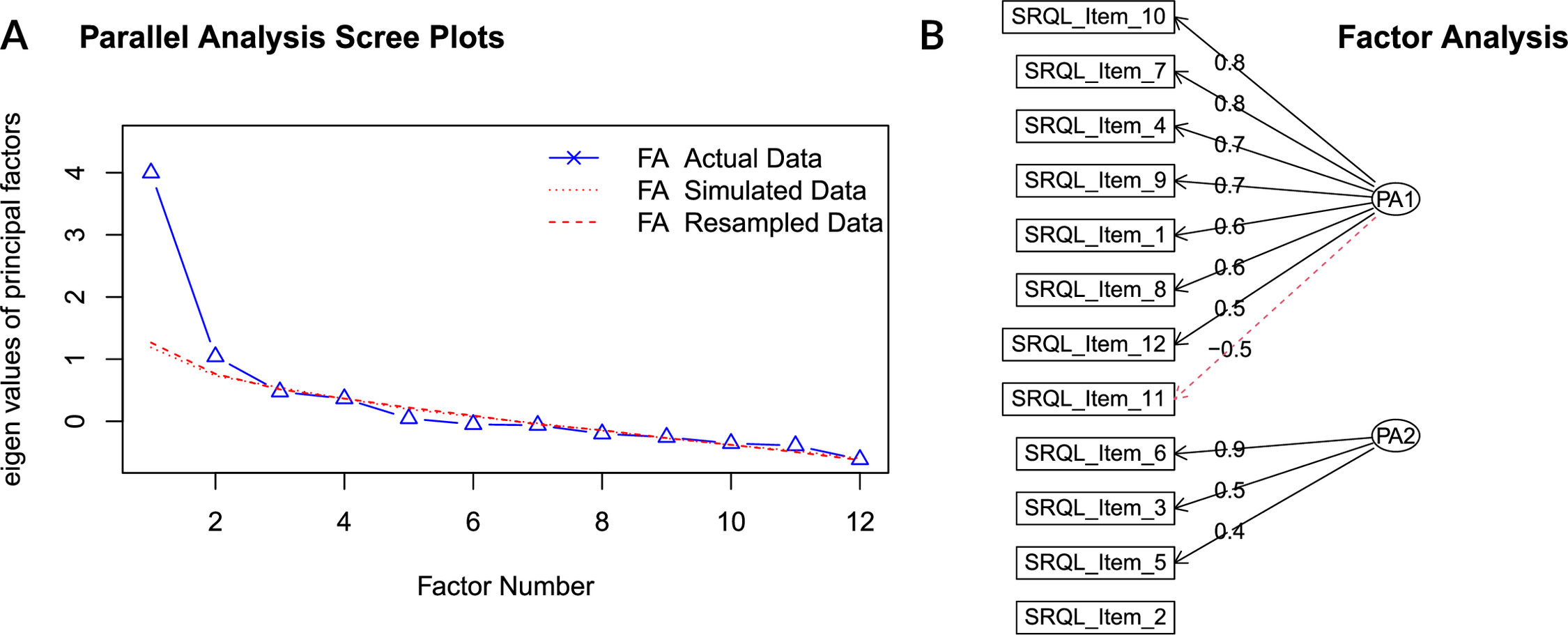

Supplement: S2 Fig — (TIF) [file pone.0331057.s009.tif]

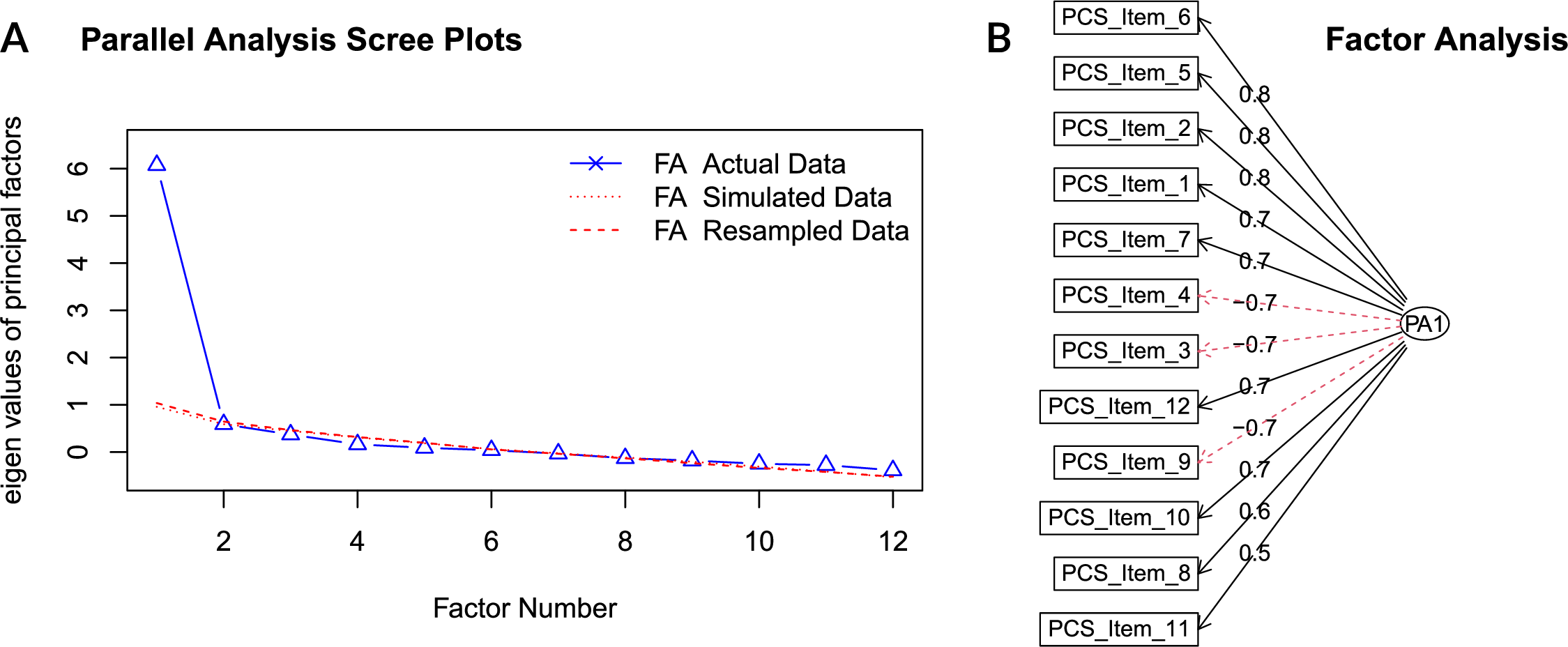

Supplement: S3 Fig — (TIF) [file pone.0331057.s010.tif]

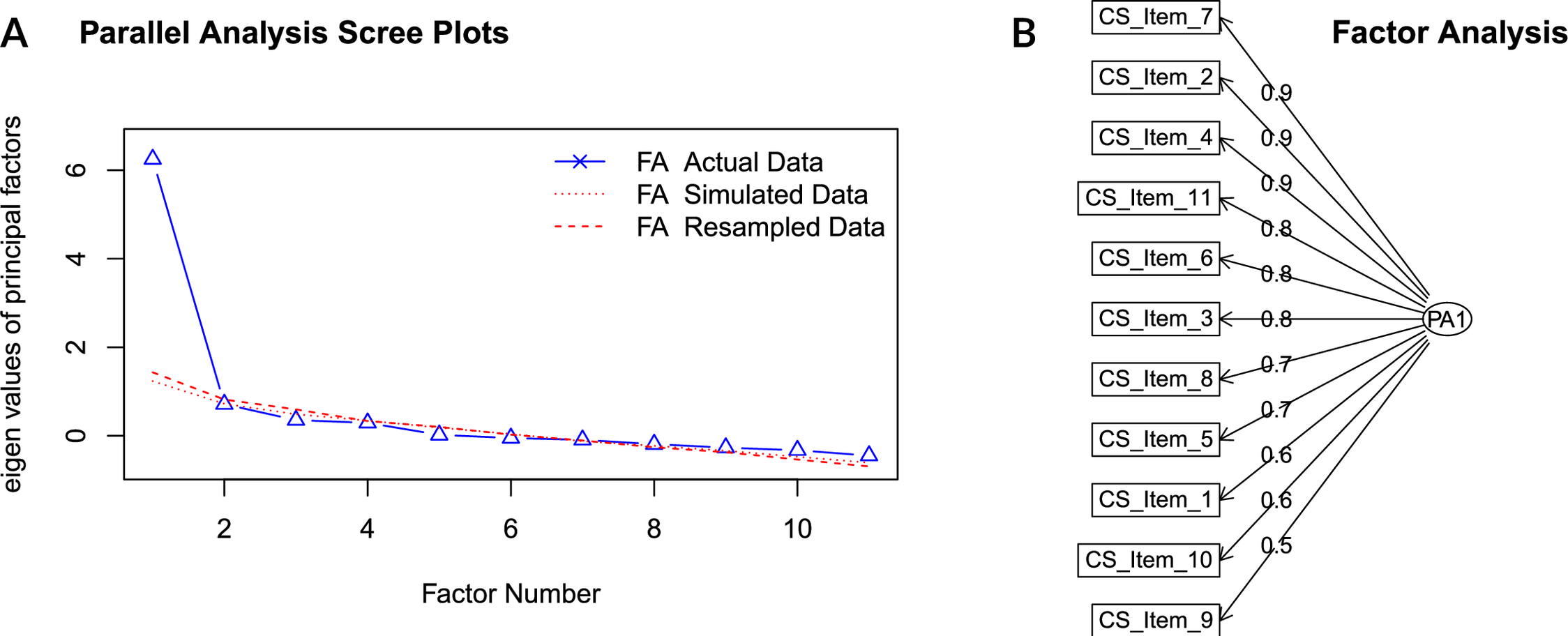

Supplement: S4 Fig — (TIF) [file pone.0331057.s011.tif]
