# Supplementary material for: Effects of mentoring on self-reflection and competence in Final year medical students’ internal medicine rotation
Source: PLoS One. 2025 Sep 2;20(9):e0331057. doi: 10.1371/journal.pone.0331057 (PMC12404468; doi:10.1371/journal.pone.0331057)
Supplement: S1 File — (DOCX) [file pone.0331057.s012.docx]

**Supplementary Material**

**SReferences**

1. Grant AM, Franklin J, Langford P. The Self-Reflection and Insight Scale: A New Measure of Private Self Conciousness. Soc Behav Pers. 2002;30(8):821–36.

2. Black AE, Deci EL. The effects of instructors’ autonomy support and students’ autonomous motivation on learning organic chemistry: A self-determination theory perspective. Sci Educ [Internet]. 2000 Nov 1;84(6):740–56. Available from: https://doi.org/10.1002/1098-237X(200011)84:6
